# Acute-onset chronic inflammatory demyelinating polyneuropathy complicating SARS-CoV-2 infection and Ad26.COV2.S vaccination: report of two cases

**DOI:** 10.1186/s41983-022-00515-4

**Published:** 2022-10-05

**Authors:** Aggeliki Fotiadou, Dimitrios Tsiptsios, Stella Karatzetzou, Sofia Kitmeridou, Ioannis Iliopoulos

**Affiliations:** Neurology Department, Democritus University of Thrace, 68100 Alexandroupolis, Greece

**Keywords:** Guillain–Barre syndrome, Chronic inflammatory demyelinating polyneuropathy, SARS-CoV-2, COVID-19

## Abstract

**Background:**

The spectrum of reported neurological sequelae associated with SARS-CoV-2 is continuously expanding, immune mediated neuropathies like Guillain–Barre syndrome (GBS) and exacerbations of preexisting chronic inflammatory demyelinating polyneuropathy (CIDP) being among them. However, respective cases of acute onset CIDP (A-CIDP) are rare.

**Case presentation:**

We hereby report two cases of A-CIDP after COVID-19 infection and Ad26.COV2.S vaccination that presented with flaccid paraparesis and acroparesthesias (Case presentation 1; female, 52) and facial diplegia accompanied by acroparesthesias (Case presentation 2; male, 62), respectively. In both instances clinical, neurophysiological and CSF findings were indicative of acute inflammatory demyelinating polyneuropathy, thus both patients were initially treated with intravenous immunoglobulins resulting in clinical improvement. Nevertheless, the first patient relapsed 5 weeks after the initial episode, thus was diagnosed with GBS with treatment related fluctuations (GBS-TRF) and treated successfully with seven plasma exchange (PLEX) sessions. However, 11 weeks from symptom onset she relapsed again. Taking into account that the second relapse occurred more than 8 weeks after the first episode, the potential diagnosis of A-CIDP was reached and oral dexamethasone 40 mg/d for 4 consecutive days every 4 weeks was administered. With regards to the second patient, he relapsed > 8 weeks after the initial episode, thus was also diagnosed with A-CIDP and treated with 7 PLEX sessions followed by similar to the aforementioned corticosteroid therapy. On 2 month follow-up both patients exhibited remarkable clinical improvement.

**Conclusions:**

Close surveillance of patients presenting with immune neuropathies in the context of SARS-CoV-2 infection or immunization is crucial for timely implementation of appropriate treatment. Prompt A-CIDP distinction from GBS-TRF is of paramount importance as treatment approach and prognosis between these two entities differ.

**Supplementary Information:**

The online version contains supplementary material available at 10.1186/s41983-022-00515-4.

## Background

Guillain–Barre syndrome (GBS) is an acute polyradiculoneuropathy of autoimmune origin typically exhibiting a monophasic course and reaching by definition its nadir within 4 weeks. Approximately 10% of these patients present with GBS with treatment related fluctuations (GBS-TRF) defined as worsening of at least one grade at GBS disability scale or by at least 5 points at MRC sum score after improvement or stabilization is achieved by immunotherapy with either Intravenous Immunoglobulins (IVIG) or Plasma Exchange (PLEX), not occurring more than 2 times and not exceeding the interval of 8 weeks from disease onset [1]. GBS’ chronic counterpart, termed chronic inflammatory demyelinating polyneuropathy (CIDP), typically exhibits a progressive or relapsing–remitting course in which the initial progressive phase by definition exceeds 8 weeks. Nevertheless, a minority of CIDP cases present with rapid deterioration within less than 8 weeks rendering distinction from GBS practically impossible at the early stage. In such instances, the term acute onset CIDP (A-CIDP) is used [2].

Immune neuropathies like GBS [3, 4] and exacerbations of preexisting CIDP have been linked to SARS-CoV-2 infection and vaccination [5]. However, respective cases of A-CIDP are rare [2, 6, 7]. We hereby report two cases of A-CIDP after COVID-19 infection and Ad26.COV2.S vaccination.

## Case presentations

### Case 1

A 52-year-old female presented with a 10 day history of progressive walking difficulties accompanied by upper and lower limb acral paresthesias. 8 weeks earlier she had made a full recovery from COVID-19 infection (confirmed by nasopharyngeal swab PCR) that did not require hospitalization. The patient had never been vaccinated against severe acute respiratory syndrome coronavirus 2 (SARS-CoV-2). On admission, she was afebrile, hemodynamically stable and required wheelchair for ambulation (GBS disability scale 4). Motor examination revealed mild upper (MRC grade 4/5) and severe lower limb (1/5) symmetrical weakness. Upper and lower limb tendon reflexes were absent bilaterally. Large-fiber and small-fiber sensory loss affecting her fingers and toes were also evident. Higher cortical, cerebellar and cranial nerve testing was unremarkable. There was no evidence of sphincter dysfunction or dysautonomia. Cerebrospinal fluid (CSF) analysis exhibited albuminocytological dissociation (protein 84 mg/dL, 0 cells). Serum and CSF testing for antiganglioside antibodies was negative. Neurophysiological evaluation (Natus UltraPro S100; Natus Medical Incorporated; USA) was suggestive of primarily demyelinating neuropathy (see Additional file 1). The constellation of clinical, CSF and neurophysiological findings were consistent with the diagnosis of acute inflammatory demyelinating polyneuropathy (AIDP). Thus, the patient was treated with a 5-day course of IVIG with subsequent improvement and ability to ambulate with double arm support at discharge (GBS disability scale 3).

5 weeks after the initial episode the patient’s symptoms recurred (GBS disability scale 4). Upper and lower limb motor nerve conduction studies revealed further deterioration (see Additional file 1). With a possible diagnosis of GBS with treatment related fluctuations (GBS-TRF), the patient underwent 7 PLEX sessions that resulted in significant improvement and ability to walk with single-arm support at discharge (GBS disability scale 3). 11 weeks from symptom onset the patient relapsed again (GBS disability scale 4). Taking into account that the second relapse occurred more than 8 weeks after the first episode, the potential diagnosis of A-CIDP was reached (Table 1). A 5-day course of IVIG was administered resulting in new improvement at discharge (GBS disability scale 3). Pulsed corticosteroid therapy with oral dexamethasone 40 mg/d for 4 consecutive days every 4 weeks was also commenced. The patient was reviewed 2 month afterwards exhibiting significant clinical improvement (GBS disability scale 1).

**Table 1:** Assessment of Patient #1 symptoms based on GBS-TRF and A-CIDP diagnostic criteria. Modified from van Doorn [8]

GBS-TRF	A-CIDP
TRF occur < 2 months from onset*	Deterioration beyond the 8th week*
Nadir reached within 8 weeks from disease onset *	Three or more relapses
Bulbar muscle weakness	Proprioception disturbance*
Autonomic instability	Absence of respiratory muscle weakness*
NCS features*	NCS features*

Key: *Present in this patient *GBS-TRF* GBS with treatment related fluctuations; *A-CIDP* Acute onset CIDP; *TRF* Treatment related fluctuations; *NCS* Nerve conduction studies

### Case 2

**Fig. 1:**
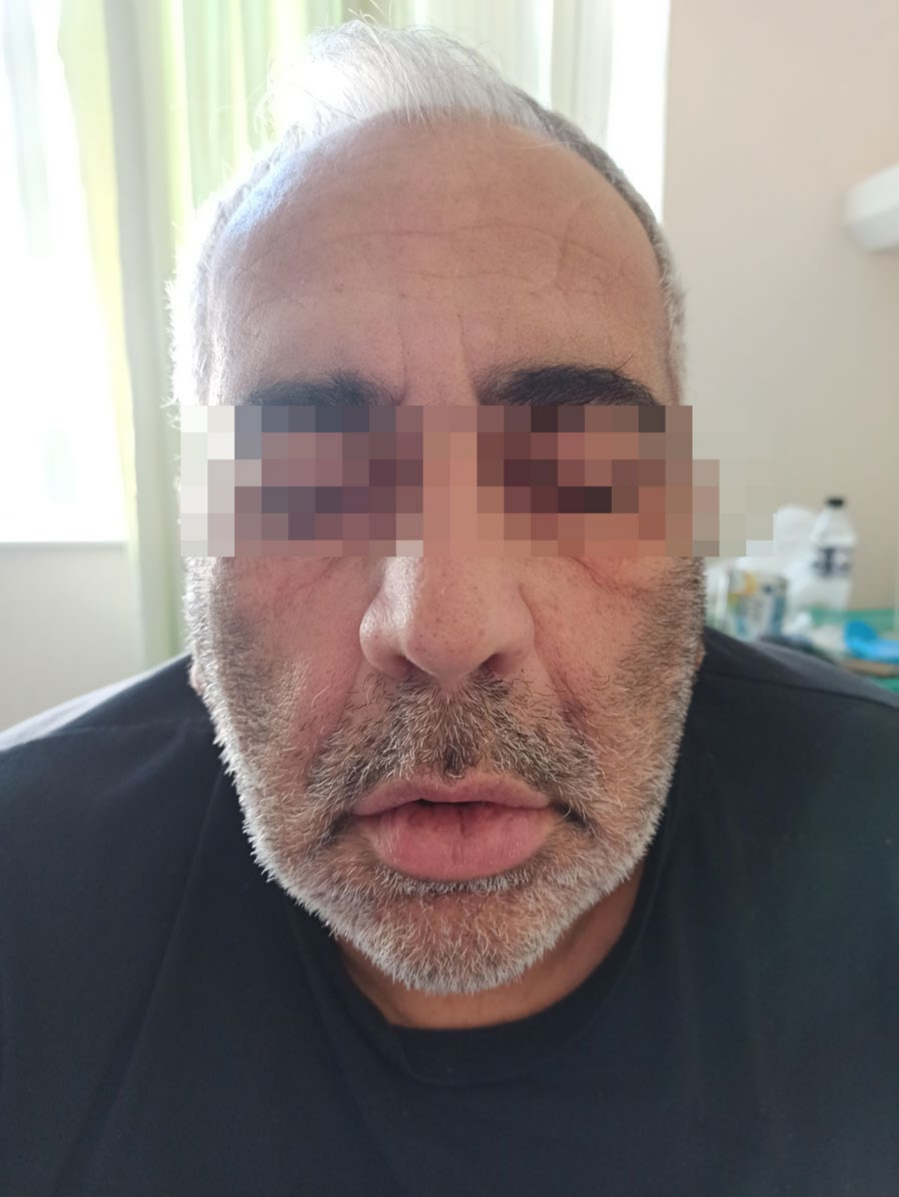
Patient exhibiting characteristic “poker face” (bilaterally absent forehead wrinkles, flattened nasolabial folds, droopy mouth corners, inability to fully close eyes, wrinkle forehead or bare teeth)

A 62-year-old male presented with an 8-day history of progressive dysarthria and walking difficulties accompanied by facial and acral paresthesias. His symptoms commenced 19 days after receiving Ad26.COV2.S vaccine. Upon admission, he was afebrile and hemodynamically stable. Nasopharyngeal swab PCR for SARS-CoV-2 was negative. He could walk unassisted, but was unable to run (GBS disability scale 2). Cranial nerve examination revealed bilateral prosopoplegia (House-Brackmann grade V) resulting in characteristic “poker face” appearance (Fig. 1; see Additional file 2). Motor examination showed mild symmetrical weakness on foot dorsiflexion (MRC grade 4/5). Achilles tendon reflexes were absent bilaterally. Large-fiber and small-fiber sensory loss affecting his feet was also noted. Higher cortical, cerebellar and the remainder cranial nerve testing was unremarkable. Sphincter dysfunction or dysautonomia were not observed. Albuminocytological dissociation was noted on CSF analysis (protein 64 mg/dL, 0 cells). Serum and CSF testing for antiganglioside antibodies was negative. Neurophysiological testing (Natus UltraPro S100; Natus Medical Incorporated; USA) was suggestive of underlying primarily demyelinating neuropathy (see Additional file 1). Based on a probable diagnosis of AIDP the patient was treated with a 5-day course of IVIG with ensuing improvement of dysesthesias and ambulation capacity, whereas bilateral facial palsy persisted (GBS disability scale 1).

58 days after the initial episode the patient’s symptoms recurred (GBS disability scale 4). Moreover, upper and lower limb motor nerve conduction studies exhibited significant deterioration (see Additional file 1). Taking into consideration that clinical nadir was reached > 8 weeks after disease onset, the patient was managed as A-CIDP (Table 2) with 7 PLEX sessions followed by pulsed corticosteroid therapy with oral dexamethasone 40 mg/d for 4 consecutive days every 4 weeks with subsequent improvement at discharge (GBS disability scale 3) and remarkable recovery on 1 month follow-up (GBS disability scale 1), even though facial diplegia persisted.

**Table 2:** Assessment of Patient #2 symptoms based on GBS-TRF and A-CIDP diagnostic criteria. Modified from van Doorn [8]

GBS-TRF	A-CIDP
TRF occur < 2 months from onset*	Deterioration beyond the 8th week*
Nadir reached within 8 weeks from disease onset	Three or more relapses
Bulbar muscle weakness	Proprioception disturbance*
Autonomic instability	Absence of respiratory muscle weakness*
NCS features*	NCS features*

Key: *Present in this patient *GBS-TRF* GBS with treatment related fluctuations; *A-CIDP* Acute onset CIDP; *TRF* Treatment related fluctuations; *NCS* Nerve conduction studies

## Discussion

Vaccination is an effective strategy in reducing the risk of acquiring COVID-19 infection and related morbidity and mortality. Nevertheless, neurological adverse events such as GBS and CIDP post-vaccination are being reported. Bifacial paresis with paresthesias is a well-described GBS phenotype in temporal association with SARS-CoV-2 vaccination [9]. The proposed mechanism is generation of cross-reactive antibodies to peripheral myelin proteins in response to SARS-CoV-2 spike protein or adenovirus vector components. The first documented cases of A-CIDP with bifacial weakness following SARS-CoV-2 vaccination were reported by Bagella and colleagues [6] and de Souza and colleagues [7]. In opposition to our second case that had been vaccinated with Ad26.COV2.S, theirs had received the first dose of ChAdOx1 nCoV-19 vaccine instead. Thus, to our knowledge ours represents the first reported case of A-CIDP manifesting with bilateral prosopoplegia related to antecedent Ad26.COV2.S vaccination.

With regards to A-CIDP associated with COVID-19 infection, the first documented case was reported by Suri and colleagues [2]. In contrast to our first case, their patient had also received the first dose of ChAdOx1 nCoV-19 vaccine 17 days prior to symptom onset. Thus, it is unclear whether A-CIDP was triggered by COVID-19 infection or ChAdOx1 nCoV-19 vaccine. To the best of our knowledge ours is the first reported case of A-CIDP solely associated with SARS-CoV-2 infection.

Prompt GBS-TRF distinction from A-CIDP is of paramount importance as treatment approach and prognosis between these two entities differ. More specifically, GBS-TRF is managed with repeated IVIG courses or PLEX, whereas A-CIDP requires long-term maintenance treatment with corticosteroids, IVIG, or PLEX with or without immunosuppressants. Rather disturbingly, in clinical practice this is not always easy. For example, there are no differences in CSF protein level and number of cells in CSF between GBS-TRF and A-CIDP. Moreover, it is proposed that patients with A-CIDP generally exhibit no cranial nerve dysfunction and are less severely disabled being able to walk independently at nadir of different deteriorations compared to patients with GBS-TRF [1]. However, both of our patients displayed significant walking difficulties and our 62 year old male severe bilateral prosopoplegia, as well. de Souza and colleagues [7] also observed that patients with A-CIDP following the Ad26.COV2.S vaccine present with bifacial paralysis and generally a more severe clinical phenotype at initial presentation that may mimic GBS, rendering early clinically distinction between COVID-19 infection or vaccination related GBS-TRF from A-CIDP practically impossible.

Identification of a biomarker of GBS-TRF or A-CIDP would enable early diagnosis and would therefore facilitate therapeutic decisions. Patients with GBS-TRF more frequently have IgM and IgG reactivity against antigangliosides as compared to the patients with A-CIDP [1]. However, antiganglioside antibodies’ sensitivity is low, as they are present in less than half of GBS patients [10]. So far, conventional nerve conduction studies have also been unable to differentiate A-CIDP from AIDP patients in initial stages. Interestingly, Sung and colleagues [11] proposed that nerve excitability tests parameters, such as superexcitability and threshold electrotonus, may be potentially useful indices to distinguish between patients with AIDP and A-CIDP. Unfortunately, nerve excitability techniques are only available in few neurophysiological departments worldwide.

## Conclusions

In keeping with available literature [6, 7], our cases also exhibit that, in contrast to classic A-CIDP [1], patients with A-CIDP related to COVID-19 infection or vaccination usually display a more severe clinical phenotype characterized by walking difficulties and/or prosopoplegia. Long-term follow-up of initially diagnosed COVID-19 related GBS patients is highly recommended, as in few instances A-CIDP and not GBS-TRF may be the correct diagnosis. To date highly specific clinical, biochemical or neurophysiological biomarkers that could differentiate COVID-19 related GBS-TRF from A-CIDP in early stages are missing.

## Supplementary Information

**Additional file 1:** Neurophysiological findings. **Table S1.** Patient 1: Initial neurophysiological findings. **Table S2.** Patient 1: Follow-up neurophysiological findings (1 month afterwards). **Table S3.** Patient 2: Initial neurophysiological findings. **Table S4.** Patient 2: Follow-up neurophysiological findings (1 month afterwards).

**Additional file 2:** Video of Patient 2 presenting with facial diplegia.

## Abbreviations

AIDP: Acute inflammatory demyelinating polyneuropathy
CSF: Cerebrospinal fluid
CIDP: Chronic inflammatory demyelinating polyneuropathy
GBS: Guillain–Barre syndrome
GBS-TRF: GBS with treatment related fluctuations
A-CIDP: Acute onset CIDP
IVIG: Intravenous Immunoglobulin
PLEX: Plasma Exchange
SARS-CoV-2: Severe acute respiratory syndrome coronavirus 2
