# Supplementary material for: Acute-onset chronic inflammatory demyelinating polyneuropathy complicating SARS-CoV-2 infection and Ad26.COV2.S vaccination: report of two cases
Source: Egypt J Neurol Psychiatr Neurosurg. 2022 Oct 5;58(1):116. doi: 10.1186/s41983-022-00515-4 (PMC9532814; doi:10.1186/s41983-022-00515-4)
Supplement: Supplementary file 1 — Additional file 1: Neurophysiological findings. Table S1. Patient 1: Initial neurophysiological findings. Table S2. Patient 1: Follow-up neurophysiological findings (1 month afterwards). Table S3. Patient 2: Initial neurophysiological findings. Table S4. Patient 2: Follow-up neurophysiological findings (1 month afterwards). [file 41983_2022_515_MOESM1_ESM.docx]

**Table 1** Patient 1: Initial neurophysiological findings

| **Sensory nerve conduction studies** | | | | | | | | | | | | |
| --- | --- | --- | --- | --- | --- | --- | --- | --- | --- | --- | --- | --- |
| **Nerve and Site** | **Onset Latency** | | **Peak**  **Latency** | **Amplitude** | | **Segment** | | **Nerve and Site** | | **Onset Latency** | **Peak**  **Latency** | **Amplitude** |
| ***Right Superficial Peroneal*** | | | | | |  | | ***Left Superficial Peroneal*** | | | | |
| Ankle | **NR** | | | | | Dorsum of foot-Ankle | | Ankle | | **NR** | | |
| ***Right* *Sural*** | | | | | |  | | ***Left* *Sural*** | | | | |
| Ankle | **NR** | | | | | Lateral foot -Ankle | | Ankle | | **NR** | | |
| ***Right Median*** | | | | | |  | | ***Left Median*** | | | | |
| Wrist | **NR** | | | | | Wrist – Digit III | | Wrist | | **NR** | | |
| ***Right Ulnar*** | | | | | |  | | ***Left Ulnar*** | | | | |
| Wrist | **NR** | | | | | Wrist- Digit V | | Wrist | | **NR** | | |
| **Motor nerve conduction studies** | | | | | | | | | | | | |
| **Nerve and Site** | | **Distal Latency** | | | **Amplitude** | | **Segment** | | **Conduction**  **Velocity** | | | **F-latency** |
| ***Right Peroneal*** | | | | | | | | | | | | |
| Ankle | | **39.8 ms**  (N.V. <6.5 ms) | | | **200 μV**  (N.V. >2 mV) | | Extensor digitorum brevis-Ankle | | **14.6 m/s**  (N.V. >40 m/s) | | |  |
| Below Fibular Head | | 61 ms | | | **110 μV** | | Ankle-Below Fibular head | |  |  |  |  |
| ***Right Tibial*** | | | | | | | | | | | | |
| Ankle | | **43.4 ms**  (N.V. <5.8 ms) | | | **400 μV**  (N.V. >5 mV) | | Abductor hallucis-Ankle | | **12.1 m/s**  (N.V. >40 m/s) | | | **NR** |
| Popliteal Fossa | | 74.1 ms | | | **170 uV** | |  | |  |  |  |  |
| ***Left Median*** | | | | | | | | | | | | |
| Wrist | | **24 ms**  (N.V. <4.4 ms) | | | **700 μV**  (N.V. >5 mV) | | Wrist-Abductor pollicis brevis | | **20.4 m/s**  (N.V. >50 m/s) | | |  |
| Elbow | | 33.3 ms | | | **510 μV** | | Elbow -Wrist | |  |  |  |  |
| ***Left Ulnar*** | | | | | | | | | | | | |
| Wrist | | **9.85 ms**  (N.V. <3.3 ms) | | | **1.34 mV**  (N.V. >5 mV) | | Wrist-Abductor digiti minimi | | **18.5 m/s**  (N.V. >50 m/s) | | | **NR** |
| Below Elbow | | 22.3 ms | | | 1.00 mV | | Below elbow - Wrist | |  |  |  |  |
| ***Right Median*** | | | | | | | | | | | | |
| Wrist | | **22.5 ms**  (N.V. <4.4 ms) | | | **1.09 mV**  (N.V.>5mV) | | Wrist-Abductor pollicis brevis | | **19.0 m/s**  (N.V. >50 m/s) | | | **NR** |
| Elbow | | 33 ms | | | **730 uV** | | Elbow -Wrist | |  |  |  |  |
| ***Right Ulnar*** | | | | | | | | | | | | |
| Wrist | | **13.45 ms**  (N.V. <3.3 ms) | | | **1.25 mV**  (N.V. >5mV) | | Wrist-Abductor digiti minimi | | **22.1 m/s**  (N.V. >50 m/s) | | | **NR** |
| Below Elbow | | 24.3 ms | | | **830 μV** | | Below elbow - Wrist | |  |  |  |  |

Abbreviations: ms: millisecond, m/s: meter per second, μV: microVolt, mV: milliVolt, NR: No Response, N.V.: Normal Values

**Table 2** Patient 1: Follow-up neurophysiological findings (1 month afterwards)

| **Motor nerve conduction studies** | | | | | |
| --- | --- | --- | --- | --- | --- |
| **Nerve and Site** | **Distal Latency** | **Amplitude** | **Segment** | **Conduction**  **Velocity** | **F-latency** |
| ***Right Peroneal*** | | | | | |
| Ankle | **NR** | | Extensor digitorum brevis-Ankle |  |  |
| Below Fibular Head | **NR** | | Ankle-Below Fibular head |  |  |
| ***Right Tibial*** | | | | | |
| Ankle | **NR** | | Abductor hallucis-Ankle |  |  |
| ***Left Median*** | | | | | |
| Wrist | **35.1 ms**  (N.V. <4.4 ms) | **540 μV**  (N.V. >5 mV) | Wrist-Abductor pollicis brevis | **8.5 m/s**  (N.V. >50 m/s) | **NR** |
| Elbow | 58.5 ms | **270 μV** | Elbow -Wrist |  |  |
| ***Left Ulnar*** | | | | | |
| Wrist | **23.8 ms**  (N.V. <3.3 ms) | **1.14 μV**  (N.V. >5 mV) | Wrist-Abductor digiti minimi | **7.1 m/s**  (N.V. >50 m/s) | **NR** |
| Below Elbow | 56 ms | **570 μV** | Below elbow - Wrist |  |  |

Abbreviations: ms: millisecond, m/s: meter per second, μV: microVolt, mV: milliVolt, NR: No Response, N.V.: Normal Values

**Table 3** Patient 2: Initial neurophysiological findings

| **Sensory nerve conduction studies** | | | | | | | | | |
| --- | --- | --- | --- | --- | --- | --- | --- | --- | --- |
| **Nerve and Site** | **Onset Latency** | | **Amplitude** | | **Conduction Velocity** | | **Segment** | | |
| ***Right Superficial Peroneal*** | | | | | | |  | | |
| Ankle | **NR** | | | | | | Dorsum of foot-Ankle | | |
| **Right *Sural*** | | | | | | |  | | |
| Ankle | **NR** | | | | | | Lateral foot -Ankle | | |
| ***Right Median*** | | | | | | |  | | |
| Wrist | 4.02 ms | | **2.8 μV**  (N.V. >10 μV) | | **34.8 m/s**  (N.V. >50 m/s) | | Wrist – Digit III | | |
| ***Right Ulnar*** | | | | | | |  | | |
| Wrist | 2.83 ms | | 11.1  **μV**  (N.V. >10 μV) | | **45.9 m/s**  (N.V. >50 m/s) | | Wrist- Digit V | | |
| **Motor nerve conduction studies** | | | | | | | | | |
| **Nerve and Site** | | **Distal Latency** | | **Amplitude** | | **Segment** | | **Conduction**  **Velocity** | **F-latency** |
| ***Right Peroneal*** | | | | | | | | | |
| Ankle | | 6 ms  (N.V. <6.5 ms) | | **550 μV**  (N.V. >2 mV) | | Extensor digitorum brevis-Ankle | | 40.2 m/s  (N.V. >40 m/s) |  |
| Below Fibular Head | | 15.25 ms | | **530 μV** | | Ankle-Below Fibular head | |  |  |
| ***Right Tibial*** | | | | | | | | | |
| Ankle | | **9.9 ms**  (N.V. <5.8 ms) | | **1.08 mV**  (N.V. >5 mV) | | Abductor hallucis-Ankle | | 40 m/s  (N.V. >40 m/s) | **NR** |
| Popliteal Fossa | | 23 ms | | **940 μV** | |  | |  |  |
| ***Right Median*** | | | | | | | | | |
| Wrist | | **5.37 ms**  (N.V. <4.4 ms) | | 5.62 mV  (N.V.>5mV) | | Wrist-Abductor pollicis brevis | | **41.7 m/s**  (N.V. >50 m/s) | **34.5 ms**  (N.V. < 30 ms) |
| Elbow | | 11.61 ms | | 3.26 mV | | Elbow -Wrist | |  |  |
| ***Right Ulnar*** | | | | | | | | | |
| Wrist | | 3.36 ms  (N.V. <3.3 ms) | | 6.91 mV  (N.V. >5mV) | | Wrist-Abductor digiti minimi | | 54.4 m/s  (N.V. >50 m/s) | 29.9 ms  (N.V. < 30 ms) |
| Below Elbow | | 8.58 ms | | 5.66 mV | | Below elbow - Wrist | |  |  |

Abbreviations: ms: millisecond, m/s: meter per second, μV: microVolt, mV: milliVolt, NR: No Response, N.V.: Normal Values

**Table 4** Patient 2: Follow-up neurophysiological findings (1 month afterwards)

| **Sensory nerve conduction studies** | | | | | | | | | | | | |
| --- | --- | --- | --- | --- | --- | --- | --- | --- | --- | --- | --- | --- |
| **Nerve and Site** | **Onset Latency** | | **Amplitude** | **Conduction Velocity** | | **Segment** | | **Nerve and Site** | | **Onset Latency** | **Amplitude** | **Conduction Velocity** |
| ***Right Superficial Peroneal*** | | | | | |  | | ***Left Superficial Peroneal*** | | | | |
| Ankle | **NR** | | | | | Dorsum of foot-Ankle | | Ankle | | **NR** | | |
| ***Right* *Sural*** | | | | | |  | | ***Left* *Sural*** | | | | |
| Ankle | **NR** | | | | | Lateral foot -Ankle | | Ankle | | **NR** | | |
| ***Right Median*** | | | | | |  | | ***Left Median*** | | | | |
| Wrist | **NR** | | | | | Wrist – Digit III | | Wrist | | **NR** | | |
| ***Right Ulnar*** | | | | | |  | | ***Left Ulnar*** | | | | |
| Wrist | **NR** | | | | | Wrist- Digit V | | Wrist | | **NR** | | |
| **Motor nerve conduction studies** | | | | | | | | | | | | |
| **Nerve and Site** | | **Distal Latency** | | | **Amplitude** | | **Segment** | | **Conduction**  **Velocity** | | | **F-latency** |
| ***Right Peroneal*** | | | | | | | | | | | | |
| Ankle | | **NR** | | | **NR** | | Extensor digitorum brevis-Ankle | |  | | |  |
| ***Right Tibial*** | | | | | | | | | | | | |
| Ankle | | **NR** | | | **NR** | | Abductor hallucis-Ankle | |  | | |  |
| ***Left Median*** | | | | | | | | | | | | |
| Wrist | | **10.95 ms**  (N.V. <4.4 ms) | | | 5.04 mV  (N.V. >5 mV) | | Wrist-Abductor pollicis brevis | | **28.6 m/s**  (N.V. >50 m/s) | | | **58.45 ms**  (N.V. < 30 ms) |
| Elbow | | 18.65 ms | | | 2.84 mV | | Elbow -Wrist | |  |  |  |  |
| ***Left Ulnar*** | | | | | | | | | | | | |
| Wrist | | **6.45 ms**  (N.V. <3.3 ms) | | | 6.08 mV  (N.V. >5 mV) | | Wrist-Abductor digiti minimi | | **29.7 m/s**  (N.V. >50 m/s) | | | **58.85 ms**  (N.V. < 30 ms) |
| Below Elbow | | 14.2 ms | | | 4.30 mV | | Below elbow - Wrist | |  |  |  |  |
| Above elbow | | 19 ms | | | 4.30 mV | | Above elbow- Below elbow | | **25.0 m/s** | | |  |
| ***Right Median*** | | | | | | | | | | | | |
| Wrist | | **10.4 ms**  (N.V. <4.4 ms) | | | 5.11 mV  (N.V.>5mV) | | Wrist-Abductor pollicis brevis | | **25.4 m/s**  (N.V. >50 m/s) | | | **59.65 ms**  (N.V. < 30 ms) |
| Elbow | | 19.25 ms | | | 3.51 mV | | Elbow -Wrist | |  |  |  |  |
| ***Right Ulnar*** | | | | | | | | | | | | |
| Wrist | | **5.25 ms**  (N.V. <3.3 ms) | | | 6.81 mV  (N.V. >5mV) | | Wrist-Abductor digiti minimi | | **23.1 m/s**  (N.V. >50 m/s) | | | **59.9 ms**  (N.V. < 30 ms) |
| Below Elbow | | 15.05 ms | | | 4.36 mV | | Below elbow - Wrist | |  |  |  |  |
| Above elbow | | 19.15 ms | | | 4.30 mV | | Above elbow- Below elbow | | **24.0 m/s** | | |  |

Abbreviations: ms: millisecond, m/s: meter per second, μV: microVolt, mV: milliVolt, NR: No Response, N.V.: Normal Values
